# Protracted Wakefulness

**Published:** 1845-09-01

**Authors:** 


*Protracted Wakefulness.*—The readers of the Boston Medical and Surgical Journal will recollect, that several years ago, a correspondent of that Journal, named Rob. F. Gourlay, represented himself as having lived without sleep for several years, with the exception of two or three occasions, when Morpheus vouchsafed a little indulgence. In the number of the Journal for Aug. 6th we observe another communication, in which he affirms he has had but one night's sleep for the six years and seven months previous. That he is honest in this assertion we are disposed to think, in the first place, because if the editor did not think so, he would probably decline his communication; and, in the second place, because we hold it to be a golden rule in the regulation of our judgment of others, always to adopt the most charitable conclusions. Assuming that the person is honest this is certainly a curious case of monomania. We take it that it must be an instance of self-deception, for it is against all physiological principles and experience, that sleep can be dispensed with for a fraction of the period he claims to have been exempted. The experiment was once made by (if we mistake not) Frederick the Great, of Prussia, and some of his officers. By the aid of stimulants and anti-soporifics they succeeded in maintaining watchfulness for a few days, but they were finally compelled to acknowledge, that if they were invincible to their martial enemies, they were not to the agreeable necessities of nature.

It is not difficult to conceive of self-deception on this subject; for we often see it exhibited in our patients for a single night or more; but that it should continue for months and years is novel indeed. We suppose that this individual may have been originally troubled with wakefulness, and have had an unconsciousness of sleep, until, either from this or other mental causes, the nervous system has become disturbed, and he is, in fact, on this topic insane.

The case is an interesting one; we wish some of our Athenian brethren would look into it, and report upon it.

